# The Role of IONM in Reducing the Occurrence of Shoulder Syndrome Following Lateral Neck Dissection for Thyroid Cancer

**DOI:** 10.3390/jcm10184246

**Published:** 2021-09-18

**Authors:** Andrea Polistena, Monia Ranalli, Stefano Avenia, Roberta Lucchini, Alessandro Sanguinetti, Sergio Galasse, Fabio Rondelli, Jacopo Vannucci, Renato Patrone, Nunzio Velotti, Giovanni Conzo, Nicola Avenia

**Affiliations:** 1 Endocrine Surgery, Santa Maria University Hospital, Perugia University, 05100 Terni, Italy; r.lucchini@aospterni.it (R.L.); a.sanguinetti@aospterni.it (A.S.); s.galasse@aospterni.it (S.G.); fabio.rondelli@unipg.it (F.R.); nicola.avenia@unipg.it (N.A.); 2 Department of Surgery Pietro Valdoni, Oncologic and Laparoscopic Surgery, Sapienza University of Rome, University Hospital Policlinico Umberto I, 00161 Rome, Italy; 3 Department of Statistical Sciences, Sapienza University of Rome, 00185 Rome, Italy; monia.ranalli@uniroma1.it; 4 Residency Programme in General Surgery, Faculty of Medicine and Surgery, University of Perugia, 06123 Perugia, Italy; stefano_avenia@libero.it; 5 Department of Surgery Paride Stefanini, Thoracic Surgery, Sapienza University of Rome, University Hospital Policlinico Umberto I, 00161 Rome, Italy; jacopo.vannucci@uniroma1.it; 6 PhD ICHT, University of Naples Federico II, 80131 Napoli, Italy; dott.patrone@gmail.com; 7 Department of Advanced Biomedical Science, University of Naples Federico II, 80131 Naples, Italy; nunzio.velotti@gmail.com; 8 Department of Traslational Medical Sciences, Division of General and Oncologic Surgery, University of Campania “Luigi Vanvitelli”, 80131 Naples, Italy; giovanni.conzo@unicampania.it

**Keywords:** IONM, accessory nerve, shoulder syndrome, thyroid cancer, neck dissection

## Abstract

Lateral neck dissection (LND) leads to a significant morbidity involving accessory nerve injury. Modified radical neck dissection (MRND) aims at preservation of the accessory nerve, but patients often present with negative functional outcomes after surgery. The role of neuromonitoring (IONM) in the prevention of shoulder syndrome has not yet been defined in comparison to nerve visualization only. We retrospectively analyzed 56 thyroid cancer patients who underwent MRND over a period of six years (2015–2020) in a high-volume institution. Demographic variables, type of surgical procedure, removed lymph nodes and the metastatic node ratio, pathology, adoption of IONM and shoulder functional outcome were investigated. The mean number of lymph nodes removed was 15.61, with a metastatic node ratio of 0.2745. IONM was used in 41.07% of patients, with a prevalence of 68% in the period 2017–2020. IONM adoption showed an effect on post-operative shoulder function. There were no effects in 89.29% of cases, and temporary and permanent effects in 8.93% and 1.79%, respectively. Confidence intervals and two-sample tests for equality of proportions were used when applicable. Expertise in high-volume centres and IONM during MRND seem to be correlated with a reduced prevalence of accessory nerve lesions and limited functional impairments. These results need to be confirmed by larger prospective randomized controlled trials.

## 1. Introduction

Despite a general good prognosis, with a 10-year overall survival rate greater than 90%, regional lymph node metastases are frequently present at the time of diagnosis in patients with papillary carcinomas (PTC) and in a lower proportion of patients with follicular carcinomas (FTC) [1].

The N stage in differentiated thyroid carcinomas (DTC) is an important prognostic factor [2], and lateral neck lymph node dissection (LND) of compartments II–V provides a complete disease staging and may reduce the risk of recurrence and, possibly, mortality rates [1]. Consequently, LND has a pivotal role in the multidisciplinary management of DTC [3].

ATA guidelines provide clear indications for LND in DTC [1], and also for medullary (MTC) [4], anaplastic (ATC) or poorly differentiated thyroid carcinomas (PDTC) [5], which are also potentially characterized by metastases to cervical lymph nodes and are both associated with worse prognosis compared to DTC [6,7,8,9].

Although a specific oncologic role is proven, LND may lead to a significant morbidity characterized by potential severe complications [3]. Among nervous complications following LND, the lesion of the accessory nerve is one of the most severe affecting post-operative quality of life—being responsible for shoulder syndrome, characterized by decreased neck and shoulder mobility with reduced elevation, flexion and abduction of the shoulder joint, anesthesia, numbness, neuropathic pain and dysmorphy or hypotrophy of the upper trapezius and sternocleidomastoid muscles [10,11,12,13].

Despite the introduction of modified radical neck dissection (MRND) and selective neck dissection (SND)—aiming at the preservation of the anatomical integrity of the accessory nerve—as gold standards of treatment compared to radical neck dissection (RND) and extended radical neck dissection (ERND), a considerable number of patients present with impaired functional outcomes after surgery [14,15].

According to a recent systematic review, the estimated prevalence of shoulder syndrome following different types of LND is variably reported in the literature, ranging between 94.8% and 27.9%. MRND and SND are associated with a lower rate of accessory nerve lesions and shoulder syndrome compared to RND [16].

Intraoperative monitoring (IONM) of the accessory nerve during MRND is largely adopted [17,18,19,20].

The IONM records accessory nerve electrical transmission before, during and after dissection using subdermal needle electrodes inserted into the sternocleidomastoid and trapezius muscles that are innervated by the monitored nerve. As previously shown, usually patients without an electrophysiological threshold increase do not develop a post-operative clinical impairment [17,18,19,20]. IONM contributes to accessory nerve identification and theoretically supports the preservation of nerve integrity, providing an intra-operative feedback of nerve function during dissection [17].

However, evidence in the literature for the usefulness of IONM in potentially reducing injury to the accessory nerve and for predicting postoperative function in neck dissection patients is minimal and contradictory, as highlighted in a recent systematic review, with a need for randomized controlled trials to determine whether such monitoring is a valuable surgical adjunct [17].

Similarly, the role of IONM has not yet been defined for recurrent laryngeal nerve identification during thyroidectomy and central neck dissection (CND) [21,22].

The aim of the present research is the analysis of the use of IONM during MRND in a large institutional series, focusing on the potential benefits in terms of functional outcome and prevention of shoulder syndrome.

## 2. Patients and Methods

### 2.1. Study Design

In our institution, IONM has been regularly used for thyroid surgery since 2015 and progressively for MRND with standardization since 2017. We designed the present study to compare the outcomes, in terms of shoulder syndrome occurrence, of patients undergoing MRND with or without IONM, in addition to direct visualization. This retrospective observational cohort study was performed according to the Strengthening the Reporting of OBservational studies in Epidemiology (STROBE) guidelines [23]. The study was conducted in accordance with the Declaration of Helsinki, but the protocol was not submitted to the evaluation of the Ethics Committee of Umbria region or registered as a clinical trial due to the retrospective design of the research. All patients gave their informed consent to the use of their clinical data for research purposes at the time of surgery.

### 2.2. Setting and Participants

We retrospectively analyzed 56 patients undergoing MRND as a single procedure, or combined with total thyroidectomy (TT) and/or CND, in a population of 515 patients operated on for thyroid cancer by the same surgical team, with standard surgical techniques, over a period of 6 years (January 2015–December 2020) in the Unit of Endocrine Surgery, Santa Maria University Hospital, Terni, University of Perugia, Italy— which is the referral center for endocrine surgery in the Umbria Region, Italy. Patients were divided into two groups, respectively, according to the adoption of IONM during dissection or not. Direct visualization of the accessory nerve during dissection was carried out in all patients. The use of IONM during MRND, through the observation period, depended on the preliminary completion of the learning curve for recurrent laryngeal nerve monitoring, the availability of specific electrodes for the sternocleidomastoid and trapezius muscles and any technical problems with the monitoring system. Functional outcome was considered at post-operative (p.o.) day 3 and after 6 months by clinical evaluation and electromyography (EMG), when appropriate.

Inclusion criteria considered were: patients aged ≥ 18 years, undergoing MRND type III (with preservation of the spinal accessory nerve, internal jugular vein, and sternocleidomastoid muscle) with or without IONM, on biopsy-proven thyroid cancer with indication for lymphadenectomy according to ATA guidelines [1,4,24]. Patients who underwent more extended procedures (MRND type I and II or RND), or those with unavailable data regarding accessory nerve functional outcome, were excluded. Medical records in the observational period were collected from our database and analyzed anonymously.

### 2.3. Preoperative Work Out

Preoperative work-out included blood tests, ECG, chest X-ray and neck ultrasound with preoperative fine needle aspiration cytology (FNAC) when appropriate, and neck computed tomography scans in selected cases. According to ATA Guidelines [1], indications for MRND in DTC were evidence of lateral compartment pathologic-like lymph nodes at the preoperative ultrasound (US) with cytological confirmation, or thyroglobulin presence in the washout fluid of fine-needle aspiration (FNA). Only 3 patients with confirmed diagnoses of thyroid cancer, due to a large palpable mass in the lateral compartment and US-evident features of pathologic-like lymph nodes including enlargement, loss of the fatty hilum, a rounded shape, hyperechogenicity, calcifications, and peripheral vascularity underwent MRND without FNA. For MTC, MRND was carried out only if there was evidence of lateral compartment pathologic-like lymph nodes at ultrasound, calcitonin in the washout fluid of FNA or high serum calcitonin level. Bilateral MRND was considered if the basal serum calcitonin level was greater than 200 pg/mL [4]. In ATC/PDTC, MRND was selected for suitable patients, with consideration of the local invasiveness of the tumor [24].

### 2.4. Surgical Procedure

The surgical procedure for MRND and general clinical management were carried out as previously reported by our group [3,6,25]. Briefly, an MRND type III was adopted in all patients. It consisted of the removal of lymph nodes from levels II to V with preservation of the sternocleidomastoid muscle, accessory nerve and internal jugular vein. The incision was carried out along the anterior margin of the sternocleidomastoid muscle and a J-shaped prolongation was adopted in cases of combined thyroid surgery. After the skin incision, a flap in the subplatysmal plain above the superficial layer of the deep cervical fascia was elevated to the level of the inferior border of the mandible. The external jugular vein was identified and the dissection carried out, with careful maneuvers, superficially through the fascia of the sternocleidomastoid muscle, which was elevated around the edge and onto the medial surface where the accessory nerve entered the muscle. The small vessels close to the accessory nerve were divided and all branches of the nerve were preserved. The dissection continued posteriorly along the entire length of the muscle. The internal jugular vein, which lies immediately behind the proximal portion of the nerve, was exposed and the dissection was carried upward to the level of the posterior portion of the digastric muscle. The complete identification of the accessory nerve was obtained, completing the dissection in the upper part of the surgical field after the sternocleidomastoid muscle was retracted posteriorly and the digastric muscle was pulled superiorly. The lymph nodes at level 2B located between the spinal accessory nerve and internal jugular vein were dissected. In this phase, the nerve was exposed completely from the sternocleidomastoid muscle to the internal jugular vein, dividing the tissue overlying the nerve.

Once the accessory nerve was completely exposed, the tissue lying superior and posterior to the nerve was dissected from the splenius capitis and levator scapulae muscles. Then, the accessory nerve was identified at Erb’s point, where it leaves the sternocleidomastoid muscle and courses through the posterior triangle of the neck to enter the anterior border of the trapezius muscle. The dissection proceeded while keeping the accessory nerve in view, with the removal of the fascia that still covered the posterior border of the sternocleidomastoid muscle and further from the anterior border of the trapezius muscle in a medial direction, including the lymphatic contents of the supraclavicular fossa.

During MRND the accessory nerve was always exposed and visually confirmed in both groups.

### 2.5. IONM System

In the study population, a NIM-Response 3.0 system (Medtronic, Minneapolis, MN, USA) set up for head and neck procedure/neck dissection was adopted to assess accessory nerve function during MRND. The IONM final reports were examined in order to verify the preserved or not-preserved transmission of the accessory nerve before, during and after dissection. Subdermal needle electrodes used as recording electrodes were inserted into the sternocleidomastoid and trapezius muscles. Additionally, a ground electrode and a stim return placed into the shoulder to complete the electrode setup and a monopolar stimulator probe (Medtronic, Minneapolis, MN, USA) were used during the procedure. When contemporary TT and CND were carried out, the NIM TriVantage tube (Medtronic, Minneapolis, MN, USA) was used for orotracheal intubation.

Muscle relaxant agents were avoided to keep the EMG responses of the examined muscle precisely assessable during general anesthesia. The accessory nerve was identified, usually proximal to its entrance into the sternocleidomastoid muscle, by application of a probe to deliver an electric stimulus that ranged from 1 to 2 mA, 100 ms, at 4 Hz. The correct identification of an intact nerve was confirmed through a series of audible acoustic signals that were generated by the system. Functional nerve integrity was once again confirmed during and at the end of the dissection by testing of the most proximal exposed portion of the nerve, and evaluating significant changes in M wave amplitude and waveform or an eventual threshold increase on electrophysiological monitoring after stimulation. The absence of a signal that was generated by the stimulator at any precise point along the nerve was accepted as evidence of loss of signal (LOS) and considered as a nerve injury. The troubleshooting protocol was always followed to check the IONM equipment for technical problems.

### 2.6. Variables

Demographic variables including sex and age, type of surgical procedure, side of MRND, number of removed lymph nodes and the metastatic node ratio, pathology on resected specimen and adoption of IONM during the observation period were investigated.

The primary functional outcome observed following MRND with and without IONM adoption, was evidence of any grade of shoulder syndrome which was defined at p.o. day 3 and after 6 months as regular accessory nerve function, or temporary or permanent nerve injuries by clinical examination and EMG when appropriate. Clinical evaluation of patients was performed after surgery using the Shoulder Pain and Disability Index (SPDI), for the evaluation of shoulder function, as previously reported [26]. The total SPDI score is the mean of the two subscales (Total pain score and Total disability score) and produces a total score ranging from 0 (best) to 100 (worst).

Patients with a negative clinical evaluation, presenting results not interfering with regular day life or working activity, were considered as negative for shoulder syndrome.

When clinical evaluation showed significant modifications in shoulder function, EMG confirmation was required. The degree of neurogenic involvement and the presence of spontaneous denervation potentials were investigated. Only partial axonal degeneration and total axonal degeneration were considered significant for persistent nerve lesions. All milder modifications observed at p.o. day 3 and recovered by a 6 months control period were considered to be transient nerve lesions. Between the two time points, all the involved patients received an intense rehabilitation program and corticosteroid treatment, when appropriate, as previously described [3].

### 2.7. Statistical Analysis

We analyzed the variables through descriptive statistics based on summary measures, plots and table distributions. We used Confidence Intervals (CI) and two sample tests for equality of proportions when applicable. A *p*-value < 0.05 or a confidence level equal to 0.95 was considered statistically significant. All of the data were analyzed using R statistical software (free open sources).

## 3. Results

### 3.1. Demographic and Surgical Results

**Figure 1:**
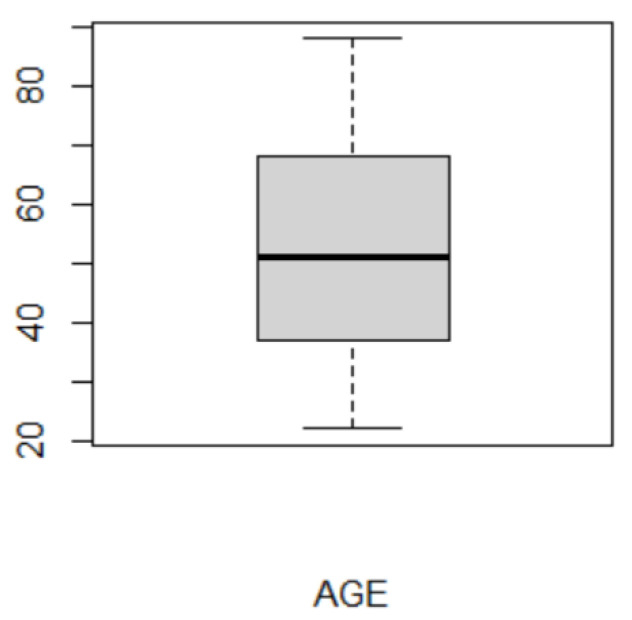
Boxplot of age distribution. Y-axis shows age in years.

The 56 cases included 30 females (53.57%) and 26 males (46.43%), with a mean age of 51.20 ± 17.59 years (Table 1). Median age was 51 years, and the corresponding interquartile range was 31 (range 22 and 88 years) (Figure 1).

**Figure 2:**
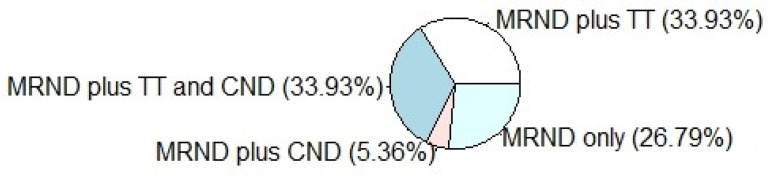
Pie chart showing the surgical procedures carried out in the series. Surgeries were classified as: 19 (33.93%) Modified Radical Neck Dissection (MRND) plus Total Thyroidectomy (TT), 19 (33.93%) MRND plus TT and Central Neck Dissection (CND), 3 (5.36%) MRND plus CND, 15 (26.79%) MRND only.

Surgical procedures carried out were classified as: 19 (33.93%) MRND plus TT, 19 (33.93%) MRND plus TT and CND, 3 (5.36%) MRND plus CND and 15 (26.79%) MRND only (Table 1, Figure 2). Among patients, left and right lymphadenectomy were equally distributed. The corresponding CI was (0.37, 0.63). It follows that the proportion was not statistically different from 0.5.

### 3.2. Lymph Node Retrieval

The mean of the number of removed lymph nodes was 15.61, while the median 14. Furthermore, the distribution seemed to be quite variable; its standard deviation was 7.85. The 95% CI was between 13.51 and 17.71. When we considered the ratio between the number of metastatic lymph nodes removed and the total number of lymph nodes, then its mean was 0.2745 with a standard deviation equal to 0.2358. The corresponding 95% CI was between 0.21 and 0.34.

### 3.3. Thyroid Cancer Subtypes

**Figure 3:**
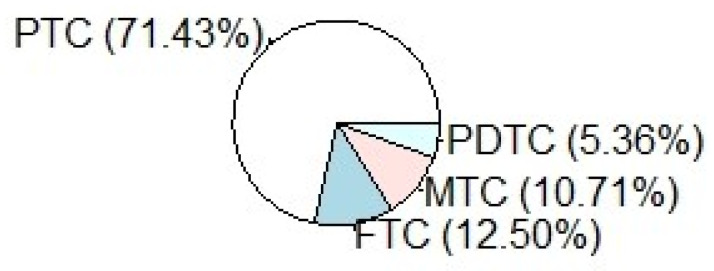
Pie Chart showing the different histotypes of cancer treated; the prevalence was 71.43% (*n* = 40) Papillary Thyroid Cancer (PTC), 12.50% (*n* = 7) Follicular Thyroid Cancer (FTC), 10.71% (*n* = 6) Medullary Thyroid Cancer (MTC) and 5.36% (*n* = 3) Poorly Differentiated Thyroid Cancer (PDTC).

Different types of cancer were observed; the prevalence was 71.43% (*n* = 40), 12.50% (*n* = 7), 10.71% (*n* = 6) and 5.36% (*n* = 3) for PTC, FTC, MTC and PDTC respectively (Table 1, Figure 3).

### 3.4. IONM Use

In the observation cohort 41.07% of surgeries were supported by the use of IONM plus direct visualization of the accessory nerve. However, its use was different over the analyzed period; indeed, between 2015–2016 the prevalence was only 19.35%, while between 2017–2020 it increased to 68%. The different prevalence observed in the two periods was statistically significant (*p* < 0.05). We conducted a two-sample test for equality of proportions, where the alternative hypothesis was two-sided and the significance level was 0.05. The CI regarding the difference of proportions, (−0.75, −0.22), showed only negative values since the increase in use of IONM was statistically significant.

### 3.5. Functional Outcome

Fifty patients out of 56 with MRND showed a post-operative SPDI at p.o. day 3 ranging between 0% and 10% and were considered negative for shoulder syndrome. All these patients maintained a low index around 0–5% at 6 months and EMG was not carried out. Six patients instead presented with significant increases on the SPDI following MRND at p.o. day 3 (Table 2) and were considered positive for a nervous lesion, with clinical evidence of shoulder syndrome. Two patients in the IONM plus visualization group who presented modification in the waveform and increases in the threshold of stimulation during surgery, and three patients in the visualization-only group, presented with significant improvement after 6 months, and the accessory nerve impairment was considered to be transient. Only one patient in the visualization-only group maintained a high SPDI at 6 months, attesting to a permanent nerve impairment with residual shoulder syndrome. EMG was carried out in the above six patients and showed at p.o. day 3, for those with transient impairment, denervation potentials referable to neuropraxia, which recovered at 6 months with normal EMG findings. The only patient with persistent nervous impairment presented EMG features of axonotmesis, almost stable at 6 months follow-up (Table 2).

The increase of IONM use during MRND seemed to have also had a mild effect on post-operative shoulder function. Overall, there were no effects in 89.29% (50) of cases, whereas transient and permanent effects were observed in 8.93% (*n* = 5) and 1.79% (*n* = 1) of patients, respectively. The distribution of the post-operative effects with IONM plus visualization or visualization only was summarized in Table 3.

According to Table 3, dependence was shown between the post-operative functional outcomes and the use of IONM; indeed, the conditional distributions were different. Furthermore, IONM decreased the incidence of shoulder syndrome, while direct visualization only, without IONM, increased both temporary and permanent nerve damage. However, statistical significance could not be analyzed since the number of observations was limited. To assess statistical significance, a larger sample size would have been needed. Indeed, considering three categories and fixing the significance level to 0.05, 108 and 141 units would have been needed to guarantee a power of 0.8 and 0.9, respectively, with a medium effect size (0.3)—and with a smaller difference, an even larger sample size. Unfortunately, since a relatively recent adoption of IONM during MRND in our institution, a proper number of cases was not available—thus attesting, for the present research, to the value of a pilot study for a larger future analysis.

When considering the increased adoption of IONM in more recent years and its supposed value in supporting the identification and preservation of the accessory nerve during MRND, we expected a progressive reduction of the complications rate over the observation time. Despite the analysis of the accessory nerve lesion rates between the different years of observation, as similarly detected when comparing the rates in the two groups, statistical significance could not be assessed, since the number of observations was limited and the events were spread over the whole period.

## 4. Discussion

Our data showed that the use of IONM during MRND contributed to the containment of accessory nerve impairment, responsible for the clinical outcome of shoulder syndrome. In our experience, IONM adoption compared to direct visualization only, although not significantly, was associated to less temporary and permanent nerve lesions; indeed in recent years, it has become a standard procedure associated with MRND in the treatment of lateral neck lymph node metastases in our institution. However, statistical significance in our series could not be assessed, since the number of observations was limited—at least in the observation time. This is one of the main limits of the research. The prevalence of accessory nerve lesions observed was 10.7%, which could be considered quite low compared to data reported in the literature. A recent systematic review with metanalysis by Larsen et al. [16] studied the prevalence of nerve injuries following neck dissection and found a 33% prevalence of accessory nerve injuries after MRND.

The analysis reported a wide range of prevalence ranging from 1.3% to more than 80%. This spread distribution might be related to many factors; the included studies were published in different years (1981–2017), and this might reflect major changes in oncological treatments and the different adoption of new technologies such as neuromonitoring, which was not considered as a variable potentially affecting the results of the metanalysis. These factors could have a major impact on the number of nerve injuries reported and should be taken into consideration.

Furthermore, the series analyzed were significantly different with regards to the sample size of the studies. The main factor which might have affected a so large distribution of prevalence, relates to the modality of diagnosis of the nerve lesion. Some authors adopted standard EMG evaluation, others only clinical examination, or both. In our analysis, the low prevalence of accessory nerve lesions reported might be attributed to many factors. First of all, the size of the examined population, which was quite limited. Secondly, the modality of accessory nerve impairment was based on clinical evaluation in all patients and EMG was used only in those presenting a modification in the post-operative clinical score; this might imply that some minor nerve injuries which remained almost asymptomatic, and not evident with only clinical examination, were lost. According to this consideration, a systematic neuro-physio-pathologic evaluation by EMG would always be beneficial in determining a more realistic prevalence of accessory nerve dysfunction following MRND in future studies. Another criticism of the present series is that we reported, despite a large distribution, a not considerably high mean of lymph nodes removed compared to other studies [27,28,29].

This might reflect that some of the procedures considered and retrospectively classified, based on the surgical procedure code attributed in the clinical records, as MRND, should have been more properly considered as SND, with less lymph nodes excised, and most probably not including level II and V, which are at higher risk of accessory nerve iatrogenic injury [14,30].

Nevertheless, this bias was systematically spread over all the patients in the series, including both approaches, IONM and visualization only during MRND, thus not affecting the evidence that IONM seemed to decrease the eventuality of shoulder syndrome, while direct visualization only, without IONM, was associated, in our experience, with both temporary and permanent nerve injuries. On the other hand, the low prevalence of accessory nerve injuries observed in our series was certainly related to the large number of patients with thyroid cancer and neck metastases treated in our institution, which is a referral center for endocrine surgery [3].

Indeed, it has been proven that, in thyroid surgery, as in all surgical fields, morbidity is inversely related to the volume of patients treated, due to the increased expertise and the adequate technologies adopted [31,32,33].

The use of IONM requires an appropriate learning curve, which may optimize the clinical benefit of this device—mostly due to a more effective interpretation of the electrophysiological response during dissection—to prevent and eventually correct inappropriate maneuvers, as often experienced in recurrent laryngeal nerve dissection with IONM during thyroidectomy [34].

Another review by Gane et al. [15] examined the prevalence and incidence of shoulder and neck dysfunction after neck dissection and identified the risk factors for post-operative complications.

The authors showed an incidence of reduced shoulder active range of motion varying from 5% to 20%, but also observed a prevalence of reduced neck active range of motion, and prevalence rates for shoulder pain, following MRND of 1–13% and of 0–100%, respectively. Again, clinical outcomes depended on the surgery carried out and on the modality of dysfunction measure used.

It is widely accepted that MRND, also known as functional neck dissection, is generally associated with considerably less morbidity, and for this reason, considering similar oncologic results, it also largely replaced RND and ERND in advanced disease [18,19,20,21,22,23,24,25,26,27,28,29,30,31,32,33,34,35,36].

Although the anatomical integrity of the accessory nerve is always supposed to be preserved following MRND, functional impairments are frequently reported, with a relevant number of patients complaining of at least chronic shoulder pain [16,37]. For this reason, during MRND, the accessory nerve should be preserved with careful dissection, avoiding even traction, potential thermal injury, extensive skeletonization and devascularization [3]. As previously shown, patients without an electrophysiological threshold increase usually do not develop a post-operative clinical impairment [19].

Evidence on the effective role of IONM in MRND are limited in the literature [17], with few prospective studies [19,20] and only one randomized trial [18] with a limited number of patients supporting the predictive value of IONM for determining shoulder function deterioration and activity restriction scores.

A fundamental point in shoulder impairment evaluation is the timing of the follow-up. Indeed, it is important to consider that shoulder function may improve 6 to 12 months after nerve-sparing operations [18]; thus, even prolonged clinical manifestations might recover with a longer follow-up period, in later controls—whereas in the literature the follow-up period is usually not standardized [16], and in our study a longer follow-up would also have been beneficial.

Another important issue is to quantify the real clinical impact of electrophysiological impairment observed post-operatively. Actually, some patients with insignificant IONM changes have a good functional prognosis and they may not present with a significant clinical counterpart due to a minor deterioration in shoulder function, and their activity restriction scores begin to improve earlier compared to those with poor prognostic findings on IONM [18].

Among different types of nerve injuries, neurotmesis and axonotmesis present with a worse prognosis, while neurapraxia, attributed to nerve motor fibers demyelination, results in short-term dysfunction, and usually recovers by remyelination within 6 to 8 weeks [11].

Furthermore, the function of trapezius muscle is often supported by an active motor branch from the cervical plexus, which may provide adequate vicarious innervation following accessory nerve injury without evident clinical effects on shoulder function [38]. Thus, as we experienced, clinically the majority of patients with neck dissection do not show shoulder movement deficits prior to discharge from the hospital because of the latent effects on trapezius muscle innervation that follow axonotmesis [11].

This can induce a delay in prompt rehabilitation programs and might affect the real estimate of shoulder function impairment, as probably occurred in our observation.

Again, EMG can help in detecting different degrees of nerve dysfunction, and it is recommended in the follow-up evaluation. An intensive program of rehabilitation with specific physiotherapy and physical therapy can improve shoulder function [11,18,39,40,41,42], as we also experienced in our current analysis and in a previous series [3], and should always be recommended [40].

Furthermore, the clinical impact of nerve injuries is also associated with a significant economic burden [43,44], with a necessity for future investigations on health-technology-assessment (HTA) and cost-effectiveness analysis, as already carried out for thyroid surgery [34]. Finally, a medico-legal issue has to be considered when dealing with technologies which might improve surgical outcomes. IONM at least provides a clinical, objective evaluation of nerve function during the surgical procedure and it certifies, despite possible functional outcomes, the correct identification and dissection of the accessory nerve, when a threshold increase in the final report is not shown [45].

## 5. Conclusions

Adoption of MRND as a standard of treatment for lateral lymph node metastases, appropriate surgical technique, expertise in high volume centres and IONM seem to be correlated with a reduced prevalence of accessory nerve lesions and consequent contained functional impairment [2,19,46,47,48].

Definitive evidence for the usefulness of IONM in reducing the prevalence of accessory nerve injury or as a method of predicting post-operative shoulder impairment outcomes following neck dissection is inconclusive at the moment. Although large prospective randomized controlled trials are required to determine the real impact of IONM in MRND, several experiences, including the results of the present research, support a potential benefit during dissection and show a correlation with improved functional outcomes.

## Figures and Tables

**Table 1:** Patient demographic and clinical characteristics. Modified Radical Neck Dissection (MRND), Total Thyroidectomy (TT), Central Neck Dissection (CND), Papillary Thyroid Cancer (PTC), Follicular Thyroid Cancer (FTC), Medullary Thyroid Cancer (MTC), Poorly Differentiated Thyroid Cancer (PDTC).

	Years, Mean ± SD
Age	51.20 ± 17.59
	n (%)
Gender	
Female	30 (53.57)
Male	26 (46.43)
Surgical procedure	
MRND + TT	19 (33.93)
MRND + TT + CND	19 (33.93)
MRND + CND	3 (5.36)
MRND	15 (26.79)
Side of the procedure	
Right	28 (50)
Left	28 (50)
Thyroid cancer subtype	
PTC	40 (71.43)
FTC	7 (12.50)
MTC	6 (10.71)
PDTC	3 (5.36)

**Table 2:** Shoulder Pain and Disability Index (SPDI) in patients with temporary and permanent lesion of the accessory nerve following MRND with or without IONM. The index is reported as total SPDI score. The total SPDI score is the mean of the two subscales (Total pain score and Total disability score) and produces a total score ranging from 0 (best) to 100 (worst).

SPDI	SPDI	
p.o. day 3	6 Months	Type of Nerve Lesion
IONM+ visualization (*n* = 2)		
57%	4%	transient
60%	5%	transient
Visualization only (*n* = 4)		
60%	10%	transient
38%	0%	transient
55%	6%	transient
75%	60%	permanent

**Table 3:** Post-operative effects distribution with IONM + visualization and visualization only and the unconditional distribution (overall).

Nerve Identification and Monitoring	No Lesion (*n*)	Temporary Lesion (*n*)	Permanent Lesion (*n*)
IONM+visualization	91.3% (21)	8.7% (2)	0% (0)
Visualization only	87.88% (29)	9.09% (3)	3.03% (1)
Overall	89.29% (50)	8.93% (5)	1.79% (1)

## Data Availability

Data are available from the corresponding author upon request.
